# Single Balloon Enteroscopy for Endoscopic Retrograde Cholangiography in a Patient with Hepaticojejunostomy after Liver Transplant

**DOI:** 10.1155/2010/701696

**Published:** 2010-05-05

**Authors:** Marta Di Pisa, Roberto Miraglia, Riccardo Volpes, Salvatore Gruttadauria, Mario Traina

**Affiliations:** ^1^Gastroenterology Service, IsMeTT-UPMC, via Tricomi 1, 90100 Palermo, Italy; ^2^Radiology Department, IsMeTT-UPMC, via Tricomi 1, 90100 Palermo, Italy; ^3^Hepatology Department, IsMeTT-UPMC, via Tricomi 1, 90100 Palermo, Italy; ^4^Surgery Department, IsMeTT-UPMC, via Tricomi 1, 90100 Palermo, Italy

## Abstract

We report a case of a post-transplant patient with hepaticojejunostomy in whom we used a single balloon enteroscopy to access the biliary tree. This procedure seems to be safe and feasible for approaching the biliary anastomosis by means of the overtube and fixation of the small bowel by the balloon.

## 1. Introduction

Biliary complications after orthotopic liver transplantation are common and constitute a relevant clinical problem (8%–50%) [1, 2]. Endoscopic retrograde cholangiopancreatography (ERCP) remains the gold standard as a therapeutic option (with a success rate of around 80%) [3–9], though this approach is feasible only in the presence of a choledocho-choledochal anastomosis in order to easily access the Vater papilla. In contrast, in patients with hepaticojejunostomy, the endoscopic approach is technically very difficult, and it is sometimes impossible to reach the biliary tree. In this condition, percutaneous transhepatic access or laparoscopic or open surgery is therefore the only feasible approach, though both these procedures are invasive and associated with high rates of morbidity [10–12]. We report a case of a posttransplant patient with hepaticojejunostomy in whom we used a single balloon enteroscopy to access the biliary tree.

## 2. Case Report

**Figure 1:**
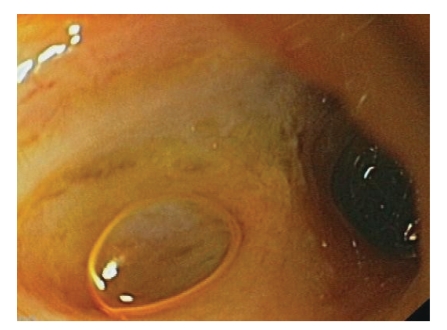
The biliodigestive anastomosis is endoscopically visualized.

A 67-year-old man underwent orthotopic liver transplantation for HCV-related cirrhosis in 2000, with a Roux-en-Y hepaticojejunostomy as biliary anastomosis. No vascular abnormalities were present on Doppler ultrasound examination. Two years later, a 6-month unsuccessful course of antiviral treatment for HCV recurrence was undertaken. Other comorbidities at that time were severe obesity, and ischemic cardiomiopathy with severe interventricular anterior artery (IVA) stenosis, treated by angioplasty. The patient was recently admitted to our institute because of the onset of severe cholestatic jaundice. At admission, physical examination showed severe obesity (BMI > 30), and no signs of hydrosaline retention. Laboratory data were the following: AST/ALT 50/54 U/L (normal: 5–40/65 U/L), bilirubin tot/dir 15.96/12.52 mg/dL (0–1.5 mg/dL), alkaline phosphates 748 U/L (40–134 U/L), and gamma-GT 290 U/L (5–85 U/L). First, a percutaneous transhepatic cholangiography (PTC) was performed. Under general anesthesia in an angiographic room, under sonographic and fluoroscopic control, a peripheral right biliary duct was punctured with a 20 G needle. A cholangiogram showed some right lobe biliary duct dilation, with multiple filling defects inside. A 10 Fr external ring catheter was positioned in a peripheric biliary duct, and a mild amount of bile and pus was drained. The biliodigestive anastomosis was not visualized. A suspicion of a missing bile duct was raised. For this reason an Endoscopic Retrograde Cholangiography (ERC) using a single balloon enteroscopy was attempted in order to visualize the anastomosis. Under general anesthesia, the patient was monitored continuously with electrocardiography, pulse oximeter, and automatic recording of blood pressure and pulse. Intravenous antibiotic prophylaxis was given before the procedure. A video enteroscope with an outer diameter of 8.5 mm and a distal balloon attached (Video-enteroscope Olympus SIF-Q180, EXERA II, Olympus Corporation, Tokyo, Japan) was passed through a balloon-attached overtube (ST SB1 overtube Olympus, Tokyo, Japan). The enteroscope was then advanced retrogradely through the duodenum, jejunum, and the leg of Roux-en-Y with the push-and-pull method. Once the anastomosis was located, the overtube was advanced towards the tip of the scope, and the balloon was inflated. The enteroscope was then advanced into the afferent loop and, once 20 cm inside the loop, the overtube balloon was deflated and advanced towards the tip of the scope. These push-and-pull maneuvers were repeated until the pouch of the afferent limb was visualized. The biliodigestive anastomosis was visualized (Figure 1). 

**Figure 2:**
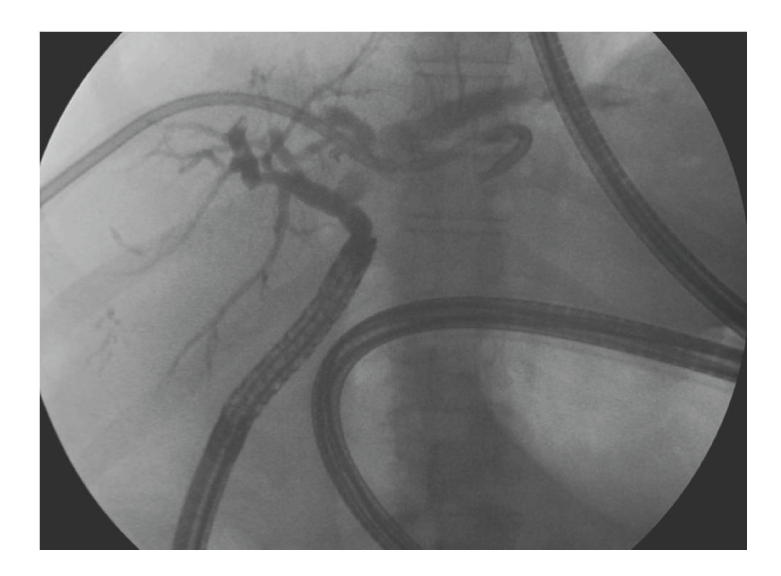
The residual biliary duct was injected and the biliary ducts visualized.

The residual biliary duct was then injected with a specific catheter for enteroscope (GT-1-TE GLO-TIP, length 320 cm, Wilson CooK). No biliary anastomosis stenosis was found (Figure 2).

A balloon catheter for stone removal by enteroscopy (EBL-18-320E, balloon catheter, double lumen, 320 cm in length, Wilson Cook, Salem, USA) was used to remove biliary sludge. Two weeks later, laboratory data showed AST/ALT 27/45 U/L (normal: 5–40/65 U/L), bilirubin tot/dir 1.56/1.24 mg/dL (0–1.5 mg/dL), alkaline phosphates 403 U/L (40–134 U/L), and gamma-GT 180 U/L (5–85 U/L). With a progressive normalization of the cholestasis, and good general conditions, the external percutaneous biliary catheter was removed.

## 3. Discussion

Biliary complications after liver transplant are common [1, 2] and, in most cases, ERCP alone is not only the best diagnostic and therapeutic treatment, with a success rate of 70–80% [3–5], but also considered the less invasive procedure, though some complications have been reported [9]. ERCP is the gold standard, in particular, for choledochocholedochal anastomosis. However, in cases of hepaticojejunostomy, ERCP might be technically very difficult and, sometimes, impossible. There are some reports in the literature on the use of double balloon enteroscopy as a safe and feasible technique for obtaining biliary access in patients with surgically altered anatomical configurations, such as those with a Roux-en-Y reconstruction. Moreover, several endoscopic techniques in patients who have undergone hepaticojejunostomy with Roux-en-Y have been used. For example, pediatric colonoscopes have been used to reach the Roux-en-Y limb in these patients. Other techniques described are balloon-assisted endoscopes or double-balloon enteroscopes [13–15]. Also, in selected cases, double-balloon enteroscopes combined with a percutaneous rendezvous technique may be helpful, as illustrated in the only case reported in the literature [16]. The use of the single balloon enteroscope for ERCP is reported only in one patient for pancreatic necrosectomy and a Roux-en-Y anastomosis, and in a second patient with chronic pancreatitis and pylorus—preserving Whipple's operation with Roux-en-Y hepaticojejunostomy [17]. To the best of our knowledge, ours is only the second report on the use of the single balloon enteroscopy in the treatment of a biliary complication after liver transplantation [18]. This procedure seems to be safe and feasible for approaching the biliary anastomosis by means of the overtube and fixation of the small bowel by the balloon. In addition, the single balloon enteroscopy has an easy maneuverability, though even limited accessories for ERCP (with a >240 cm length and 7 Fr in size, Wilson Cook, Salem, USA devices) can be used. We conclude that this diagnostic method, together with such therapeutic instruments as sphincterotomes, balloon dilation catheters, stone removal catheters, and specific stents, can be useful in enabling access to the Roux-en-Y limb in patients who develop biliary problems following surgical hepaticojejunostomy after liver transplant.
